# Role of Training and Detraining on Inflammatory and Metabolic Profile in Infarcted Rats: Influences of Cardiovascular Autonomic Nervous System

**DOI:** 10.1155/2014/207131

**Published:** 2014-06-18

**Authors:** Bruno Rodrigues, Aline Alves Santana, Aline Boveto Santamarina, Lila Missae Oyama, Érico Chagas Caperuto, Cláudio Teodoro de Souza, Catarina de Andrade Barboza, Leandro Yanase Rocha, Diego Figueroa, Cristiano Mostarda, Maria Cláudia Irigoyen, Fábio Santos Lira

**Affiliations:** ^1^Human Movement Laboratory, Sao Judas Tadeu University (USJT), Avenida Taquari 546, 03166-000 São Paulo, SP, Brazil; ^2^Physiology Department, Nutrition Physiology Discipline, Federal University of São Paulo (UNIFESP), Rua Sena Madureira 1500, 04021-001 São Paulo, SP, Brazil; ^3^Laboratory of Exercise Physiology and Biochemistry, Universidade do Extremo Sul Catarinense (UNESC), Avenida Universitária 1105, 88806-000 Criciúma, SC, Brazil; ^4^Hypertension Unit, Heart Institute (InCor), Medical School of University of São Paulo, Avenida Doutor Enéas de Carvalho Aguiar 44, 05403-900 São Paulo, SP, Brazil; ^5^Immunometabolism Research Group, Department of Physical Education, University of São Paulo State (UNESP), Rua Roberto Simonsen 305, 19060-900 Presidente Prudente, SP, Brazil

## Abstract

The aim of this study was to evaluate the effects of exercise training (ET, 50–70% of VO_2 max_, 5 days/week) and detraining (DT) on inflammatory and metabolic profile after myocardial infarction (MI) in rats. Male Wistar rats were divided into control (C, *n* = 8), sedentary infarcted (SI, *n* = 9), trained infarcted (TI, *n* = 10; 3 months of ET), and detrained infarcted (DI, *n* = 11; 2 months of ET + 1 month of DT). After ET and DT protocols, ventricular function and inflammation, cardiovascular autonomic modulation (spectral analysis), and adipose tissue inflammation and lipolytic pathway were evaluated. ET after MI improved cardiac and vascular autonomic modulation, and these benefits were correlated with reduced inflammatory cytokines on the heart and adipose tissue. These positive changes were sustained even after 1 month of detraining. No expressive changes were observed in oxidative stress and lipolytic pathway in experimental groups. In conclusion, our results strongly suggest that the autonomic improvement promoted by ET, and maintained even after the detraining period, was associated with reduced inflammatory profile in the left ventricle and adipose tissue of rats subjected to MI. These data encourage enhancing cardiovascular autonomic function as a therapeutic strategy for the treatment of inflammatory process triggered by MI.

## 1. Introduction

Cardiovascular diseases (CVD) are the leading causes of death worldwide and account for billions of dollars in hospital costs [1, 2]. Coronary artery disease with subsequent myocardial infarction (MI) is the most prevalent CVD and commonly progresses to heart failure (HF) in affected individuals [3, 4]. The inflammatory process not only promotes the onset and progression of atheroma, but also contributes decisively to several cardiac remodeling events, such as apoptosis, changes in extracellular matrix, and other structural alterations [5].

Cytokines, such as interleukin (IL-6), IL-1*β*, and tumor necrosis factor-alpha (TNF-*α*), are detected in ischemic tissue as early as 3 hours after the onset of ischemia and are involved in later deleterious responses during cardiac remodeling [6]. Additionally, several studies have suggested that adipose tissue can contribute to the inflammatory status of HF after MI [7–9]. Shimizu et al. [8] have found that deterioration of systolic cardiac function leads to impaired glucose metabolism and increased infiltration of mononuclear cells (mainly macrophages into visceral fat), and these changes, in turn, were associated with increased production of proinflammatory cytokines in HF.

The mechanism underlying immune activation after ischemia has been dealt with by several research studies, and at least five hypotheses have been proposed [10]. Thus, this issue needs further clarification. In recent years, several studies have lent strength to the hypothesis of a direct relationship between parasympathetic activation and immune system response [11–14]. In this scenario, inflammatory mediators (cytokines) produced in peripheral tissues would warn the central nervous system by a direct central action or by afferent stimulation of the vagus nerve. Given this and considering that MI may promote a chronic imbalance between sympathetic and parasympathetic nervous systems [15, 16], we might speculate that this autonomic change may be associated with increased inflammatory status after MI.

On the other hand, exercise training (ET) seems to be an important strategy in the management of MI induced changes and HF, particularly with regard to improving cardiovascular autonomic control and reducing chronic inflammatory response, which would, in turn, reduce cardiac workload, decrease the risk of fatal arrhythmias, and increase survival rates of affected individuals [17]. However, the effects of ET and detraining (DT) on cardiovascular autonomic modulation and their reflexes on cardiac and adipose tissue inflammatory profile and on lipolytic pathway remain poorly understood. The present investigation was designed to evaluate the effects of ET and DT on (i) left ventricle systolic function and inflammatory status, (ii) hemodynamic profile and cardiovascular autonomic modulation, and (iii) adipose tissue inflammation and lipolytic protein expression.

## 2. Methods

### 2.1. Animals

Experiments were performed in adult male Wistar rats (250–300 g) from the Animal House of the São Judas Tadeu University, São Paulo, Brazil. Rats were fed standard laboratory chow and water ad libitum. The animals were housed in collective polycarbonate cages in a temperature-controlled room (22-23°C) and under 54-55% humidity with a 12 h dark-light cycle (light 07:00–19:00 h). The experimental protocol was approved by the institutional animal care and use committee of the São Judas Tadeu University (008/2013), and this investigation was conducted in accordance with the Principles of Laboratory Animal Care formulated by the National Institutes of Health (National Institutes of Health Publication number 96-23, revised 1996).

The rats were randomly assigned to four groups: control (C, *n* = 8; followed up for 3 months), sedentary infarcted (SI, *n* = 9; after MI, the animals were followed up for 3 months), trained infarcted (TI, *n* = 10; after MI, the animals were trained for 3 months), and detrained infarcted (DI, *n* = 11; after MI, the animals were trained for 2 months and detrained for 1 month).

### 2.2. Myocardial Infarction

Anaesthetized rats (80 mg/kg ketamine and 12 mg/kg xylazine, i.p.) underwent surgical occlusion of the left coronary artery, which resulted in MI as described previously [18–21]. Briefly, after intubation, animals were positive-pressure ventilated with room air at 2.5 mL, 65 strokes/min with a pressure-cycled rodent ventilator (Harvard Apparatus, Model 683, Holliston, MA, USA). For induction of MI, a 2 cm left lateral thoracotomy was performed in the third intercostal space, and the left anterior descending coronary artery was occluded with a nylon (6.0) suture at approximately 1 mm from its origin below the tip of the left atrium. The C animals underwent the same procedures except that myocardial ischemia was not induced. The chest was closed with a silk suture.

### 2.3. Echocardiographic Evaluation

Initial echocardiographic evaluation was performed 2 days after MI. Final evaluation was performed after following, training, and detraining periods (1 day after final VO_2max⁡_ measurement). Echocardiographic evaluations were performed by a blinded observer, under the guidelines of the American Society of Echocardiography. Rats were anaesthetized (80 mg/kg ketamine and 12 mg/kg xylazine, i.p.), and images were obtained with a 10–14 mHz linear transducer in a SEQUOIA 512 (Acuson Corporation, Mountain View, CA, USA) for measurements of MI akinetic area, left ventricular internal diameter in systole (LVSYS), left ventricular internal diameter in diastole (LVDD), ejection fraction (EF), and velocity of circumferential fiber shortening (VCF) in experimental groups [18–20]. The MI area was delimited leading to analysis of the movement of the LV walls. Regions with systolic shortening classified as absent were considered infarcted, as described in detail elsewhere [18–20].

MI akinetic area was confirmed by histological evaluation. Hearts were transected 5 mm below coronary ligation, and transversal slices were processed and embedded in paraplast. Sections of 5 mm were stained with Picrosirius (red staining) for fibrosis evaluation. Fibrosis was evaluated semiquantitatively using a fibrosis score, ranging from 0 (no fibrotic tissue) to 4 (for scar only replacing the myocardium) in a computerized image acquisition (Leica Imaging Systems, USA), and analysis (Image Quant-Leica) [19, 20].

### 2.4. Maximal Oxygen Consumption (VO_2max_)

Sedentary, trained, and detrained groups were adapted to the treadmill (10 min/day; 0.3 km/h) for 4 days after MI. VO_2max⁡_ evaluation was performed twice, initially at 6 days after MI and again after the training and detraining protocols. This parameter was determined by analyzing expired gas during a progressive exercise ramp protocol, with 3 m/min increments every 3 min and no grade until exhaustion. Gas analysis was performed using an oxygen (S-3A/I) analyzer (Ametek, Pittsburgh, PA, USA). VO_2_ was calculated using the measured flow through the metabolic chamber, the expired fraction of effluent oxygen, and the fraction of oxygen in room air, as described elsewhere [19, 20].

### 2.5. Exercise Training and Detraining Protocols

Exercise training was performed on a motorized treadmill at moderate intensity (50–70% of VO_2max⁡_) for 1 h a day, 5 days a week, with a gradual increase in speed from 0.3 to 1.5 km/h [19, 20]. C group was placed on the treadmill every day for the same length of time as TI and DI groups, without movement. TI group was submitted to exercise training for 3 months. DI animals underwent training for 2 months and then remained sedentary, after exercise training cessation (i.e., “detraining”), for 1 month. To detrain, the rats were allowed to roam their cages freely under a 12 h dark-light cycle at 22-23°C and under 54-55% humidity, with free access to water and rodent chow [20].

### 2.6. Hemodynamic Assessment and Cardiovascular Autonomic Modulation

One day after the final echocardiographic evaluation, 2 catheters filled with 0.06 mL of saline were implanted into the femoral artery and femoral vein of the anesthetized rats (80 mg/kg ketamine and 12 mg/kg xylazine, i.p.). Twenty-four hours later, the arterial cannula was connected to a strain-gauge transducer (Blood Pressure XDCR; Kent Scientific, Torrington, CT, USA), and arterial pressure (AP) signals were recorded over a 30 min period in conscious animals by a microcomputer equipped with an analog-to-digital converter board (WinDaq, 2 kHz, DATAQ, Springfield, OH, USA). The recorded data were analyzed on a beat-to-beat basis to quantify changes in mean AP (MAP) and heart rate (HR) [19–22].

The overall variability of the pulse interval (PI) and systolic AP (SAP) in the time domain was assessed by total variance (Var IP and Var SAP) of the time series. Fluctuations in PI and SAP were further assessed in the frequency domain by means of autoregressive spectral estimation. The theoretical and analytical procedures for autoregressive modelling of oscillatory components have been described previously [21–23]. Briefly, the PI and SAP series derived from each recording were divided into 300 beat segments with a 50% overlap. The spectra of each segment were calculated via the Levinson-Durbin recursion and the order of the model chosen according to Akaike's criterion, with the oscillatory components quantified in LF (0.2–0.6 Hz) and high frequency (HF; 0.6–3.0 Hz) ranges. The normalized units were obtained by calculating the power of LF and HF correlating each to the total power, after subtracting the power of the very LF component (frequencies < 0.2 Hz). The alpha index was calculated when the magnitude of square coherence between the signals of IP and SAP exceeded 0.5 (range 0-1) in the LF band. After consistency calculation, the alpha index was extracted from the square root of the ratio between the LF of IP and LF of SAP.

### 2.7. Protein Analysis by Western Blotting

After euthanasia, the epididymal adipose tissue was rapidly removed and homogenized in 1.5 mL extraction buffer (100 mM Trizma, 1% SDS, 100 mM sodium pyrophosphate, 100 mM sodium fluoride, 10 mM EDTA and 10 mM sodium vanadate) and boiled for 10 min. The extracts were then centrifuged at 12,000 rpm at 4°C for 40 min to remove insoluble material. Protein determination in the supernatants was performed by the Bradford dye method using the Bio-Rad reagent (Bio-Rad Laboratories, Hercules, CA, USA). The proteins were treated with Laemmli sample buffer containing dithiothreitol and boiled for 5 min before loading into 10% SDS-PAGE in a Bio-Rad miniature slab gel apparatus. Electrotransfer of proteins from the gel to nitrocellulose was performed for ~1 h at 15 V (constant) in a Bio-Rad semidry transfer apparatus. Nonspecific protein binding to the nitrocellulose was reduced by preincubation for 2 h at 22°C in blocking buffer (5% nonfat dry milk, 10 mM Tris, 150 mM NaCl, and 0.02% Tween 20).

The nitrocellulose membranes were incubated overnight at 4°C with antibodies against TLR4 (toll-like receptor 4), and nuclear factor kappa B p50 (NF-kBp50), adipose triglyceride lipase (ATGL), comparative gene identification-58 (CGI58), perilipin, AKT, PKC, AMPK, glutathione peroxidase, catalase, and alpha-tubulin were obtained from Santa Cruz Biotechnology (Santa Cruz, CA, USA) diluted in blocking buffer to which 1% bovine serum album (BSA) was added and then washed for 30 min in blocking buffer without BSA. The blots were subsequently incubated with peroxidase-conjugated secondary antibody for 1 h [24].

By evaluation of protein loading, membranes were stripped and reblotted with anti-alpha-tubulin antibody, as appropriate. Specific bands were detected by chemiluminescence and visualisation/capture was performed by exposure of the membranes to RX films. Band intensities were quantified by optical densitometry of developed autoradiographs (Scion Image software, Scion Corporation, Frederick, MD, USA).

### 2.8. TNF-*α*, IL-10, and IL-6 Concentration Assessment by ELISA

Frozen epididymal adipose tissues (0.1–0.3 g) were homogenized in RIPA buffer (0.625% Nonidet P-40, 0.625% sodium deoxycholate, 6.25 mM sodium phosphate, and 1 mM ethylenediaminetetraacetic acid at pH 7.4) containing 10 *μ*g/mL of a protease inhibitor cocktail (Sigma-Aldrich, St. Louis, MO, USA). Homogenates were centrifuged at 12,000 g for 10 min at 4°C, the supernatant was saved, and protein concentration was determined using the Bradford assay (Bio-Rad, Hercules, CA, USA) with bovine serum albumin as a reference. Quantitative assessment of tumor necrosis factor-alpha (TNF-*α*), interleukin-6 (IL-6), and interleukin-10 (IL-10) proteins was carried out by ELISA (DuoSet ELISA, R&D Systems, Minneapolis, MN, USA). For the TNF-*α* (DY510), IL-6 (DY506), and IL-10 (DY522) assays, sensitivity was found to be 5.0 pg/mL in the range of 31.2–2.000 pg/mL. The intra-assay variability and interassay variability of the TNF-*α* and IL-6 kits were 2.7–5.2% and 4.9–9.5%, respectively. Assay sensitivity for IL-10 was 10 pg/mL in the range from 31.2 to 2.000 pg/mL. The intra-assay variability of the IL-10 kit was 2.0–4.2%, and its interassay variability was 3.3–6.4% [24]. All samples were run as duplicates, and the mean value was reported.

### 2.9. Statistical Analyses

Statistical analyses were performed with SPSS software (Version 20.0 for Windows; SPSS Inc., Chicago, USA). Data are reported as mean ± SEM. After confirming that all continuous variables were normally distributed using the Kolmogorov-Smirnov test, statistical differences between the groups were obtained by two-way ANOVA followed by the Bonferroni posttest. Statistical differences between the data measured over time were assessed using repeated-measures ANOVA. Pearson's correlation was used to study the association between EF and TNF-*α* and HF band of PI and IL-6 and TNF-*α* levels in the left ventricle and between LF band of SAP and TNF-*α* values in the adipose tissue. All tests were two sided and the significance level was established at *P* < 0.05.

## 3. Results

### 3.1. Animals

Body weight was similar among all studied groups at the beginning of the protocol (~273 ± 10 g). At the end of the protocol, C (501 ± 15 g), SI (433 ± 10 g), TI (490 ± 5 g), and DI (484 ± 6 g) groups displayed higher body weight when compared to their initial values. C, TI, and DI groups had higher body weight than the SI group. Epididymal adipose tissue weight was lower in infarcted rats (SI: 2.5 ± 0.3; TI: 2.7 ± 0.1; DI: 2.3 ± 0.2 g) when compared to C rats (3.6 ± 0.2 g).

Initially, after MI, VO_2max⁡_ values were lower in infarcted animals (SI: 55 ± 4; TI: 50 ± 5; DI: 52 ± 3 mL/Kg/min^−1^) when compared to C animals (85 ± 5 mL/Kg/min^−1^). After training and detraining protocols, TI (80 ± 5 mL/Kg/min^−1^) and DI (71 ± 3 mL/Kg/min^−1^) groups increased VO_2max⁡_ when compared to the SI group (57 ± 5 mL/Kg/min^−1^). Although VO_2max⁡_ was lower in DI animals when compared to C animals (84 ± 3 mL/Kg/min^−1^) and TI, it was still higher than the values found for SI animals. As to VO_2max⁡_, an interaction was found between different evaluation times and experimental groups (*F* = 74.201, *P* < 0.001).

### 3.2. LV Morphometry and Function

At the initial assessment, MI akinetic area (evaluated by echocardiography) was similar among infarcted groups (~41 ± 3%). However, ET was able to reduce MI akinetic area in TI (28 ± 3%) group when compared to SI (43 ± 4%). This reduction was sustained even after 1 month of detraining, as observed in the DI (27 ± 4%) group. In order to confirm MI akinetic area, a histological analysis of fibrosis score was carried out. TI (0.78 ± 0.07) and DI (0.86 ± 0.08) groups showed a reduction in fibrosis score in relation to the SI (2.85 ± 0.12) group. A correlation between fibrosis score and MI area as evaluated by the echocardiogram (*r* = 0.79; *P* = 0.004) was also observed.

**Figure 1:**
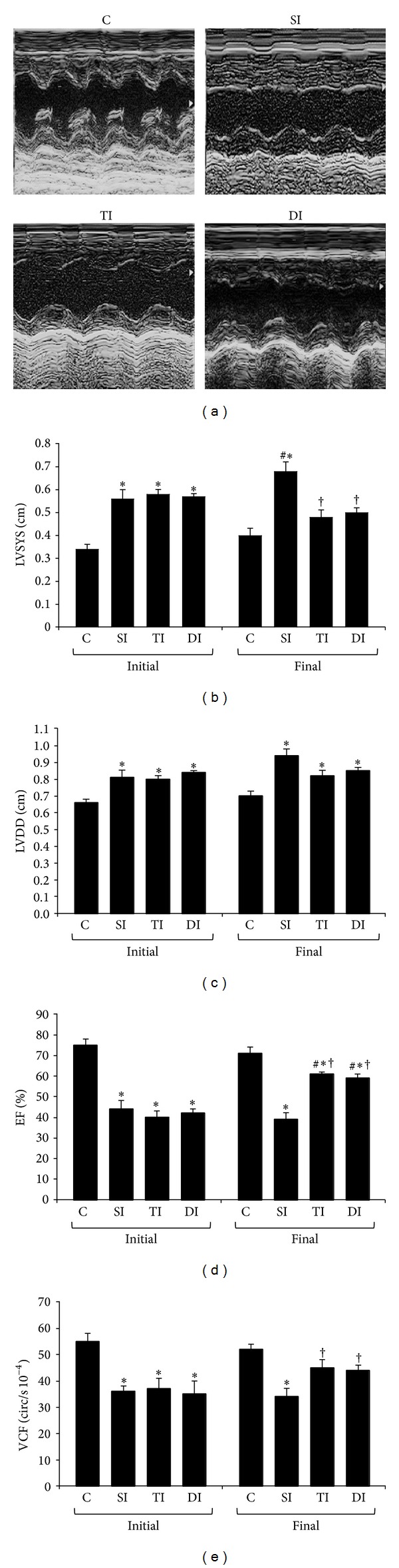
Echocardiographic assessment of LV function. (a) Representative M-mode echocardiographic images. (b) LV internal diameter in systole (LVSYS). (c) LV internal diameter in diastole (LVDD). (d) Ejection fraction (EF). (e) Velocity of circumferential fiber shortening (VCF). Quantitative echocardiographic parameters were evaluated in control (C), sedentary infarcted (SI), trained infarcted (TI), and detrained infarcted (DI) rats. ^#^ *P* < 0.05 versus initial evaluation in the same group; *P < 0.05 versus C; ^†^ *P* < 0.05 versus SI.

Representative M-mode echocardiographic images are presented in Figure 1. Regarding ventricular morphometry, SI, TI, and DI groups displayed higher LVSYS (Figure 1(b)) and LVDD (Figure 1(c)) when compared to the C group at the initial evaluation. At the final evaluation, LVSYS was lower in TI and DI animals when compared to SI animals.

Systolic function, evaluated by EF (Figure 1(d)) and VCF (Figure 1(e)), was more reduced in SI, TI, and DI groups when compared to C group at the initial evaluation. EF was higher after ET in TI and DI animals than their respective initial evaluations and when compared to SI animals. However, these final values were still lower when compared to C animals. Similarly, VCF was lower in SI rats at the final evaluation but higher in TI and DI rats when compared to SI rats. An interaction between different evaluation times and experimental groups was observed in VCF (*F* = 25.569, *P* < 0.001).

### 3.3. Hemodynamic Profile and Cardiovascular Autonomic Modulation

Hemodynamic and cardiovascular autonomic modulation data are presented in Table 1. The SI group showed lower SAP when compared to the C group. However, DAP, MAP, and HR values were similar between experimental groups. An interaction between ET and MI was also observed in SAP (*F* = 16.768; *P* = 0.001).

Regarding PI variability, Var PI, LF, and HF bands' values were impaired in SI animals, while ET was able to improve these parameters in TI animals. Furthermore, it should be noted that these cardiac benefits of ET were maintained after 1 month of detraining, as observed in DI animals (Table 1). No differences were observed in LF/HF ratio. Var SAP values were not changed by MI, training, or detraining protocols. As for the LF band of SAP, an indicator of vascular sympathetic modulation, SI group displayed an increase in these values when compared to C group. In addition, TI and DI groups showed lower values than SI group, as shown in Table 1. No differences were observed in HF band values between experimental groups. Interactions of ET and MI in Var PI (*F* = 11.090; *P* = 0.01) and absolute values of LF band (*F* = 13.141; *P* = 0.009) parameters were also found.

### 3.4. Cytokines in the Left Ventricle

**Figure 2:**
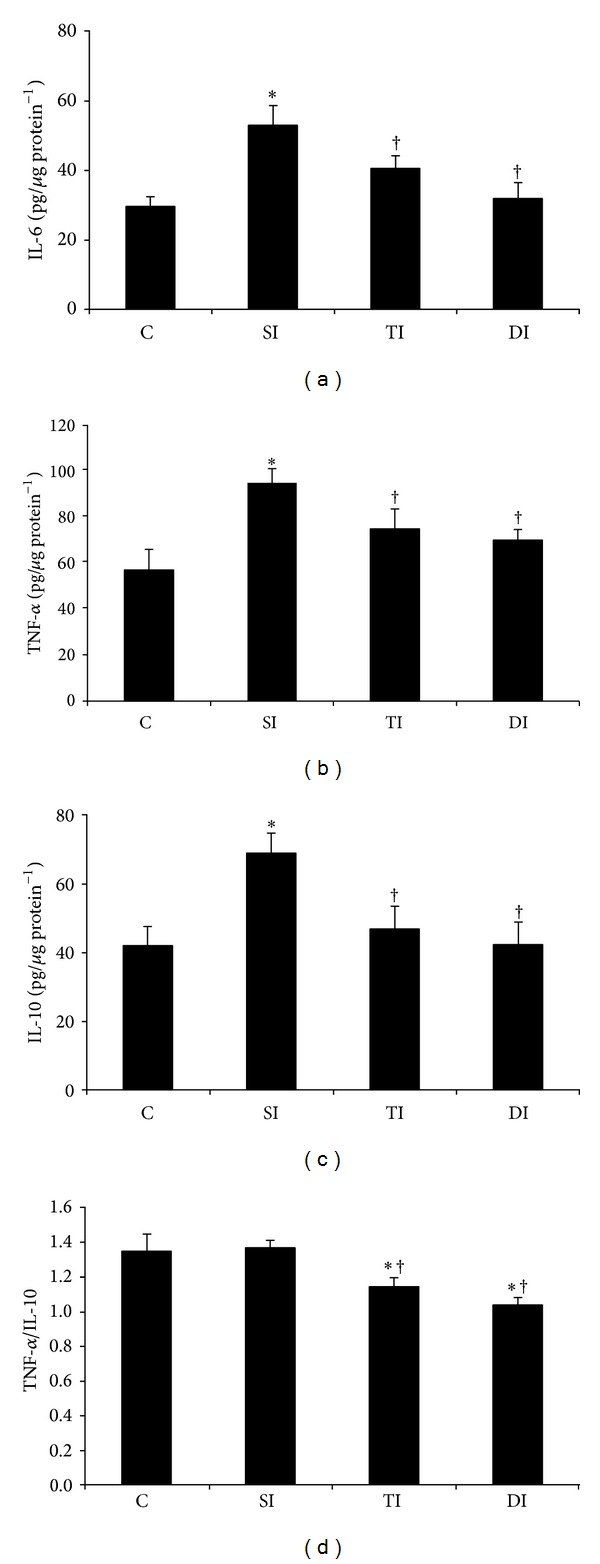
Left ventricle inflammatory profile in control (C), sedentary infarcted (SI), trained infarcted (TI), and detrained infarcted (DI) rats. *P < 0.05 versus C; ^†^ *P* < 0.05 versus SI.

IL-6 (Figure 2(a)), TNF-*α* (Figure 2(b)), and IL-10 (Figure 2(c)) concentrations in the left ventricle were higher in SI group when compared to the C group. However, TI and DI animals presented lower values when compared to SI animals. Additionally, TNF-*α*/IL-10 ratio was lower in TI and DI rats when compared to both C and SI rats (Figure 2(d)). Interactions were observed between ET and MI in IL-6 (*F* = 17.544; *P* = 0.001) and IL-10 (*F* = 19.344; *P* < 0.001) parameters.

### 3.5. Cytokines and Oxidative Stress in the Adipose Tissue

**Figure 3:**
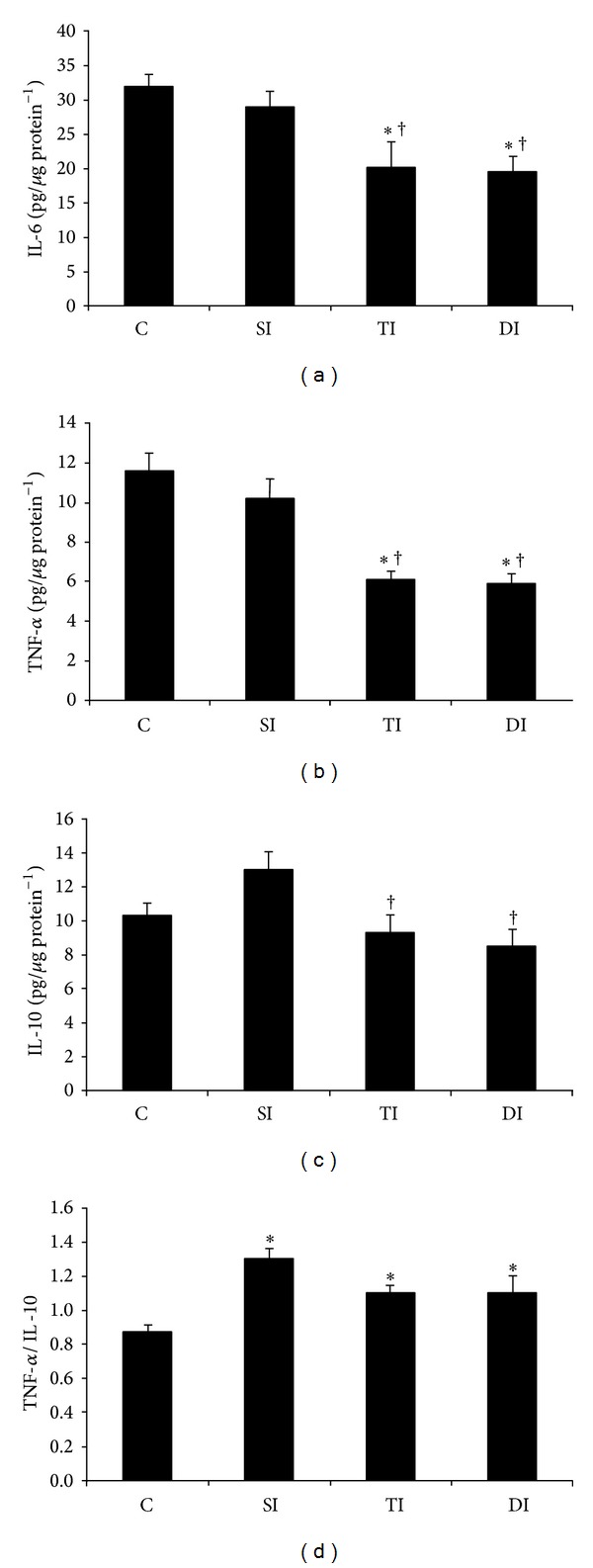
Inflammatory profile on epididymal adiposetissue of control (C), sedentary infarcted (SI), trained infarcted (TI), and detrained infarcted (DI) rats. *P < 0.05 versus C; ^†^ *P* < 0.05 versus SI.

IL-6 and TNF-*α* values were lower in TI and DI animals when compared to both C and SI animals (Figures 3(a) and 3(b)). The content of IL-10 was lower in TI and DI groups when compared to the SI group (Figure 3(c)). In this scenario, TNF-*α*/IL-10 ratio was higher in SI, TI, and DI animals when compared to C animals (Figure 3(d)).

**Figure 4:**
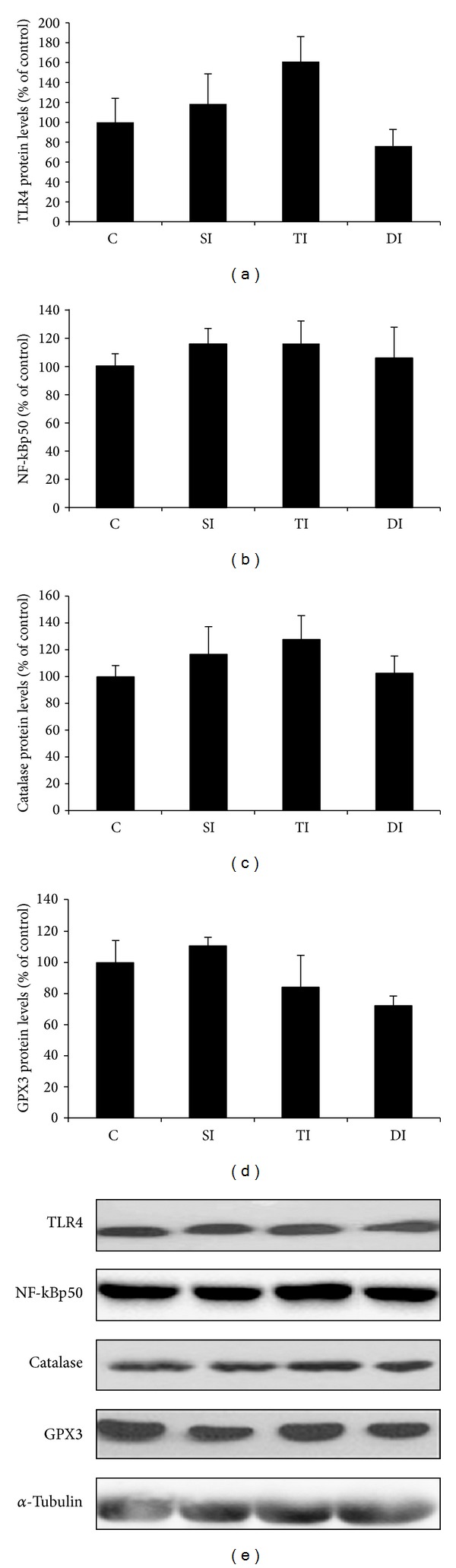
Protein levels of TLR4, NF-kBp50, catalase, and GPX3 on epididymal adiposetissue of control (C), sedentary infarcted (SI), trained infarcted (TI), and detrained infarcted (DI) rats.

In addition to these data, TLR4 (Figure 4(a)), NF-kBp50 (Figure 4(b)), catalase (Figure 4(c)), and GPX3 (Figure 4(d)) protein levels expression were analyzed. No differences in protein expression were observed between experimental groups.

### 3.6. Quantification of Lipolytic Proteins

**Figure 5:**
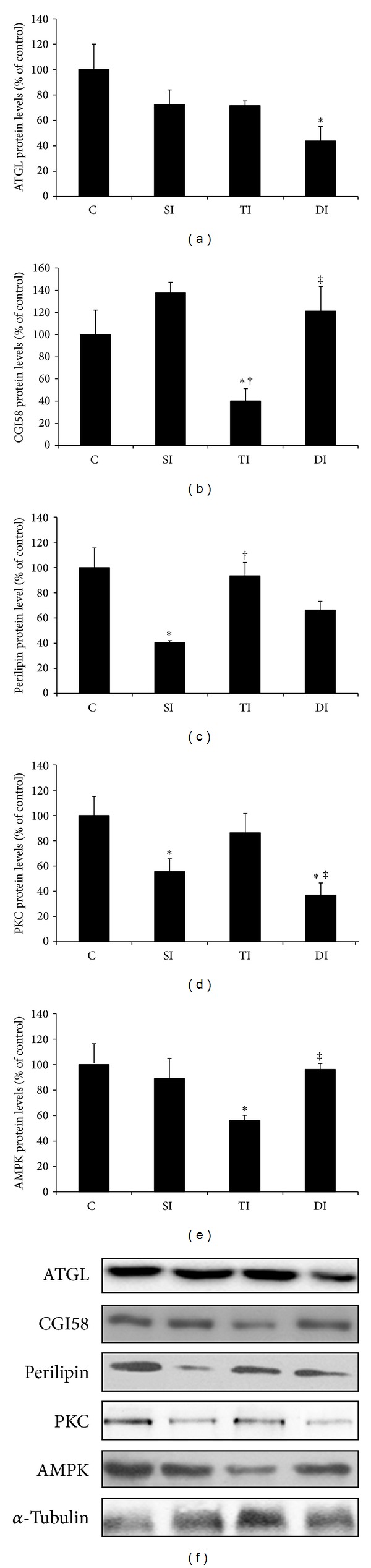
Protein levels of ATGL, CGI58, perilipin, PKC, and AMPKon epididymal adiposetissue of control (C), sedentary infarcted (SI), trained infarcted (TI), and detrained infarcted (DI) rats. *P < 0.05 versus C; ^†^ *P* < 0.05 versus SI; ^‡^ *P* < 0.05 versus TI.

ATGL protein levels were lower in DI animals when compared to C animals (Figure 5(a)). After ET period, TI rats displayed lower CGI58 expression levels when compared to C, SI, and DI rats (Figure 5(b)). Perilipin protein levels were lower in SI group when compared to those in C, while ET normalized this response, as observed in TI group (Figure 5(c)). SI and DI animals presented lower PKC protein expression when compared to C animals (Figure 5(d)). Furthermore, an additional reduction in protein levels was observed in DI rats when compared to TI rats. AMPK protein expression was lower in the adipose tissue of TI group when compared to C and DI groups (Figure 5(e)).

### 3.7. Correlations

**Figure 6:**
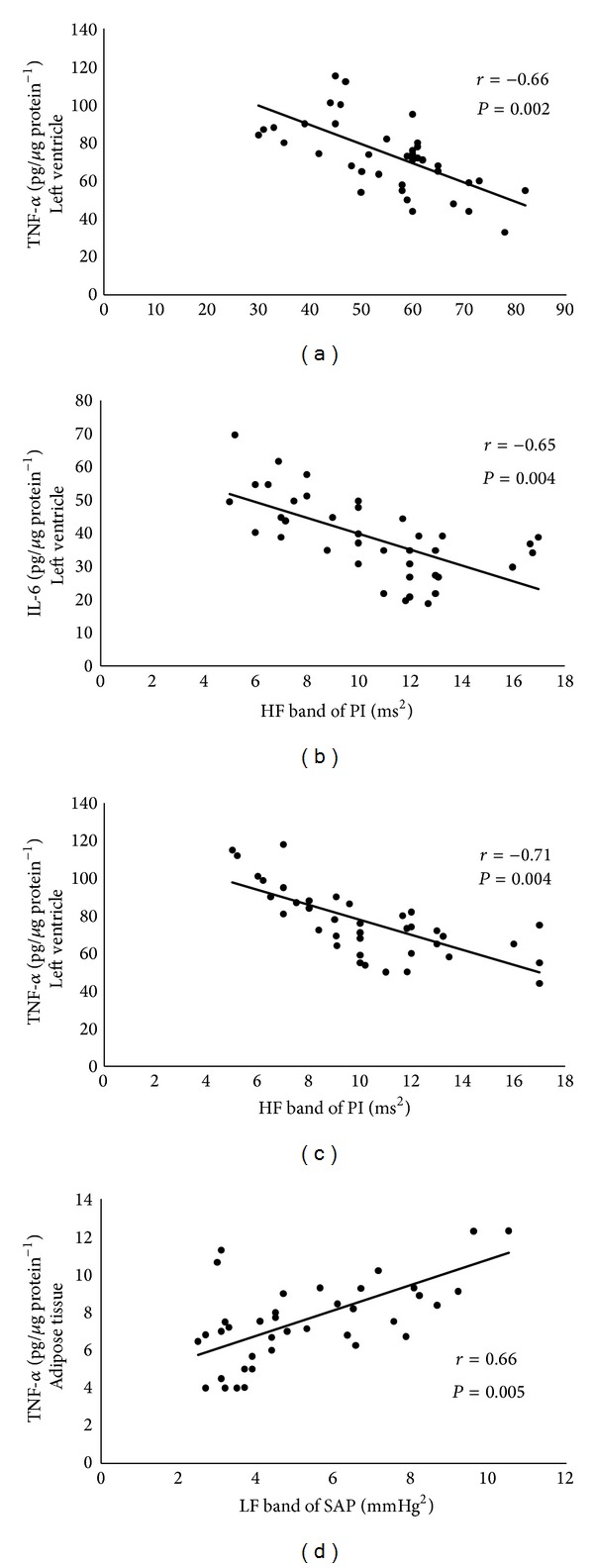
Correlations between (a) ejection fraction (EF) and TNF-*α* levels in the LV. (b) High frequency band of pulse interval variability (HF band) and IL-6 levels in the LV. (c) High frequency band of pulse interval variability (HF band) and TNF-*α* levels in the LV. (d) Low frequency band of systolic arterial pressure variability (LF band) and TNF-*α* levels in the adipose tissue.

Correlation analyses are shown in Figure 6. The improvement in EF was negatively correlated with TNF-*α* values in the left ventricle of experimental animals (Figure 6(a)). Negative correlations were observed between HF band of PI and IL-6 levels (Figure 6(b)) and TNF-*α* values (Figure 6(c)) in the left ventricle of experimental rats. Additionally, positive correlation was observed between LF band of SAP and TNF-*α* values (Figure 6(d)) in the adipose tissue of experimental rats.

## 4. Discussion

Over the past decades, significant progress has been achieved in understanding the functional and molecular derangements after MI, and this has been translated into new therapeutic approaches in this field. However, despite advances of modern cardiology and improved quality of life and survival rates for patients, MI remains the major cause of death worldwide [4]. Thus, there is a compelling need for new pharmacological and nonpharmacological approaches to improve current clinical outcomes. To address this issue, a number of studies have focused on identifying the effects of ET on cardiac, autonomic, and inflammatory process after MI, which would well work as targets for MI therapy. Indeed, given that the adipose tissue has been widely regarded as a huge gland orchestrating the biochemical processes associated with endothelial dysfunction, hypertension, atherosclerosis, and heart diseases [25], this tissue seems to be another likely ET target for MI complications.

In the present study, we found that the sedentary infarcted group exhibited lower body weight than the other groups. The weight loss observed in the infarcted model animals may lead to cardiac cachexia [26, 27]. Haehling et al. [27] have reported that weight loss in the cachectic patient predominantly affects muscle protein; however, bone and fat tissue are likewise affected later in the course of the disease. In addition, these authors have found that proinflammatory cytokines, particularly TNF-*α*, seem to play critical role in this condition. In this sense, we found that aerobic ET was able to reduce IL-6 and TNF-*α* and normalize IL-10 protein content on the epididymal adipose tissue and on the left ventricle of trained and detrained animals. These changes in the left ventricle inflammatory profile are probably associated with systolic function improvement and MI area reduction in these animals. Actually, the protective effects of ET on inflammatory markers have been widely discussed [28]. In this sense, Adamopoulos et al. [29] have demonstrated a reduction in inflammatory markers after 12 weeks of aerobic ET in patients with moderate to severe heart failure. The researchers have also suggested that the improvement of exercise tolerance was related to the attenuation of the systemic inflammatory process.

Individuals with increased risk for coronary artery disease presented reduced interleukin-1 (IL-1), IL-6, TNF-*α*, and C-reactive levels, together with improved IL-10 and transforming growth factor beta-1 systemic levels after an ET program [30]. Similarly, the association between aerobic and resistance ET decreased the concentration of TNF-*α* receptors I and II in patients with HF [31]. In an experimental model of HF, Batista Jr. et al. [32] have shown that aerobic ET in infarcted rats increased the ratio IL-10/TNF-*α* in the soleus muscle of animals, thus emphasizing the anti-inflammatory effect of exercise after an ischemic event.

Although the anti-inflammatory role of exercise has been widely acknowledged in the literature, the underlying mechanisms associated with improved inflammatory profile in patients after an ischemic event have yet to be fully understood. Recent approaches to exercise and immune function [33] have pointed out that the reduction of chronic inflammation in associated diseases is mediated, to some extent, both via a reduction of visceral fat mass and by the establishment of an anti-inflammatory environment with each bout of exercise. On the other hand, according to the “inflammatory reflex” theory proposed by Tracey [11], the nervous system, in particular the parasympathetic autonomic system, reflexively regulates the inflammatory response in real time. Thus, the activation of the vagus nerve would lead to a reduced production of cytokines, which would in turn decrease the inflammatory response in models of septic and aseptic inflammation [11].

As ET is strongly associated with positive adaptations in the autonomic nervous system, particularly with reduced sympathetic tonus and increased vagal tonus [15, 16, 19], we tested the possibility that the amelioration of cardiac and adipose tissue inflammatory profile could be related to changes in cardiac and vascular autonomic nervous system. It has been reported that chronic autonomic imbalance is a key element in the pathophysiology of heart failure after MI [15, 16]. In fact, in the present investigation, pulse interval and systolic arterial pressure variability evaluation suggest a reduced parasympathetic modulation to the heart and increased cardiac and vascular sympathetic modulation in SI animals. However, ET was able to increase vagal modulation (HF band of PI) and normalize sympathetic modulation to the heart (LF band of PI) and vessels (LF band of SAP). This suggests that ET may not only increase the reflex responses mediated by the parasympathetic nervous system, but also may suppress the impact of the sympathetic nervous system in ischemic heart disease. It is worth noting that these positive adaptations were maintained after 1 month of detraining.

Similarly, La Rovere et al. [34] have demonstrated that MI patients undergoing four weeks of ET displayed increased survival, together with an adequate modulation of the autonomic balance towards increased vagal activity. Regarding the duration of the ET beneficial effects, our group demonstrated that even after 3 and 4 weeks of detraining the autonomic and ventricular adaptations were sustained in diabetic [35] and infarcted rats [20], respectively.

The observed correlations between cardiac vagal modulation (HF band of PI) and inflammatory cytokines in the heart tissue and between vascular sympathetic modulation (LF band of SAP) and inflammatory cytokine in the adipose tissue point to the important role the autonomic nervous system may play in the inflammatory profile of infarcted rats. Accordingly, in humans, significantly reduced heart rate variability is associated with elevated levels of inflammatory cytokines (IL-6) and C-reactive protein [36]. Lanza et al. [37] have shown that serum C-reactive protein levels were significantly associated with reduced heart rate variability in patients with unstable angina. This association has also been observed in healthy individuals, patients with stable coronary artery disease, and HF [38]. However, to our knowledge, our study is the first to report the relationship between autonomic benefits of ET and their impact on the inflammatory profile of cardiac and adipose tissues of infarcted rats.

On the other hand, it has been suggested that lipolysis is induced by several cytokines, such as IL-1*β*, IL-6, and TNF-*α* [39]. However, we observed reduced adipose tissue mass in infarcted rats when compared to control group, and we found no significant increases in cytokine content in this tissue, but we did observe some alterations in lipolytic-related proteins.

Myocardial infarction significantly reduced adipose tissue mass, and this loss can be promoted, at least in part, by reduced perilipin protein expression, favoring action of lipases, particularly hormone-sensitive lipase and adipose triglyceride lipase. ATGL and HSL are able to degrade TG by cleaving the ester bond, thus governing the lipolysis pathway in adipose tissue [40]. We did not find any changes in ATGL protein expression, but we remain cautious about this particular finding, since we did not evaluate ATGL phosphorylation form. Further studies are needed for better understanding of the mechanism underlying lipolysis in the adipose tissue of infarcted rats. In another experimental model, we have found a close association between lipolysis process and inflammatory response [41]. Also, it has been known that free fatty acids produced by lipolysis in adipocytes stimulate monocytes, producing a paracrine loop between lipolysis and local inflammation. In this sense, several members of toll-like receptors (TLRs) family would be expressed in adipocytes and would then recognize the free fatty acids as ligands, thus promoting the expression of proinflammatory cytokines [42].

These findings led us to evaluate the proteins involved with the lipolysis pathway. We might then postulate that the lipolysis process may be a strong activator of inflammatory response in the adipose tissue. However, no alterations were found in protein levels in this tissue in the different studied groups. Thus, further studies are needed for fuller understanding of the mechanisms involved in the lipolytic process of MI animals and for assessing the role of ET and detraining in this condition.

In addition, several studies on obesity and cancer cachexia in experimental models have shown that the presence of infiltrating monocytes in adipose tissue promotes cytokine production and leads to low grade inflammation [43–45]. However, this condition is yet unknown in myocardial infarction.

Taken together, the findings of the current study demonstrated that (i) ET after myocardial infarction was able to improve autonomic modulation to the heart and vessels, (ii) cardiovascular autonomic modulation improvement by ET was correlated with reduced inflammatory cytokines on the heart and adipose tissue, (iii) these benefits were sustained even after one month of detraining, and (iv) no expressive changes were observed in oxidative stress and lipolytic pathway in experimental groups.

In conclusion, our results strongly suggest that the autonomic improvement promoted by ET, sustained even during the detraining period, was associated with reduced inflammatory profile in the left ventricle and adipose tissue of rats undergoing MI. Thus, our findings indicate that improving cardiovascular autonomic function may be a powerful therapeutic strategy for the treatment of the inflammatory process triggered by MI.

However, the cause-effect relationship between cardiovascular autonomic function and inflammatory profile can only be inferred so far. Other systemic and local mechanisms cannot be ruled out. Furthermore, the maintenance of the autonomic and inflammatory benefits even after a short period of detraining may be important information when planning holidays or breaks in cardiovascular rehabilitation process.

## Figures and Tables

**Table 1:** Hemodynamic evaluation, pulse interval, and systolic arterial pressure variability in control (C), sedentary infarcted (SI), trained infarcted (TI), and detrained infarcted (DI) rats.

Parameter/group	C	SI	TI	DI
SAP (mmHg)	120 ± 3	109 ± 5*	126 ± 6^†^	125 ± 4^†^
DAP (mmHg)	81 ± 3	86 ± 2	89 ± 4	86 ± 7
MAP (mmHg)	108 ± 7	93 ± 10	110 ± 4	107 ± 6
HR (bpm)	327 ± 5	334 ± 8	330 ± 7	338 ± 10
PI variability				
Var PI (ms^2^)	101 ± 13	58 ± 10*	128 ± 12^†^	127 ± 5^†^
LF (ms^2^)	4.0 ± 0.5	1.5 ± 0.2*	4.5 ± 1.0^†^	4.4 ± 0.6^†^
LF (%)	22.1 ± 1.6	19.0 ± 3.2	29.8 ± 2.2^†^	28.8 ± 2.4^†^
HF (ms^2^)	13.9 ± 0.9	6.6 ± 0.7*	10.6 ± 1.1^∗†^	10.9 ± 0.9^∗†^
HF (%)	77.8 ± 1.6	80.9 ± 3.1	70.1 ± 2.8	71.2 ± 2.9
LF/HF	0.28 ± 0.04	0.24 ± 0.03	0.42 ± 0.09	0.40 ± 0.05
SAP variability				
Vas SAP (mmHg^2^)	26.7 ± 5	20.1 ± 2	27.3 ± 3	21.4 ± 2
LF (mmHg^2^)	3.3 ± 0.3	7.0 ± 1*	3.5 ± 0.6^†^	3.9 ± 0.5^†^
HF (mmHg^2^)	2.0 ± 0.1	1.6 ± 0.3	1.9 ± 0.2	1.7 ± 0.4

Values are expressed as mean ± SEM. SAP: systolic arterial pressure; DAP: diastolic arterial pressure; MAP: mean arterial pressure; HR: heart rate; Var PI: variance of pulse interval; LF: low frequency band; HF: high frequency band; Var SAP: variance of systolic arterial pressure. *P < 0.05 versus C; ^†^ *P* < 0.05 versus SI.
